# Gender Variations and Entrepreneurial Intentions: A Cross-Sectional Analysis of Finalist Undergraduate Students at Mbarara University of Science and Technology, Uganda

**DOI:** 10.12688/f1000research.148994.2

**Published:** 2025-11-13

**Authors:** Aloysius Rukundo, Naster Tumwebembire, Wilbroad Aryatwijuka, Miriam Tugiramasiko, Specioza Twinamasiko

**Affiliations:** 1 Department of Educational Foundations and Psychology, Mbarara University of Science and Technology, Mbarara, Western Region, Uganda; 2 Faculty of Business and Management Sciences, Mbarara University of Science and Technology, Mbarara, Western Region, Uganda; 3 Directorate of Research and Graduate Training, Mbarara University of Science and Technology, Mbarara, Western Region, Uganda; 4 Faculty of Interdisciplinary Studies, Mbarara University of Science and Technology, Mbarara, Western Region, Uganda; 5 Community Engagement and Service Learning, Mbarara University of Science and Technology,, Mbarara, Western Region, Uganda

**Keywords:** Entrepreneurship, gender, socio-demographics, undergraduates, business, and intentions.

## Abstract

**Background:**

Recently, the dynamics of gender and entrepreneurship have received more attention; however, the subtleties of how gender affects entrepreneurial intentions remain a challenge, despite empowerment through skills and formal training. This study seeks to establish gender differences in entrepreneurial intentions in educational settings as well as the broader context of gender equity in achieving financial independence among finalist undergraduate students. The results of this study contribute to a better understanding of gender-related factors that influence entrepreneurial intentions among undergraduate students.

**Methodology:**

This was a cross-sectional and quantitative study. The study site was the town campus of Mbarara University of Science and Technology. The study population consisted of finalist businesses and students. The data were managed and analyzed using STATA version 17. T-tests of independent samples and means were used for data analysis.

**Results:**

Of the 404 respondents, the majority were males (56.2%). Entrepreneurial Intentions were higher among females with a history of business ownership (M = 22.7, SD = 5.1) than among those without a history of business ownership (M = 19.5, SD = 6.9), at t = 2.25, p <0.05. Further, entrepreneurial intentions were lower among male students whose male caretakers or parents had business as an occupation (M = 19.5, SD = 6.9) than their counterparts whose male parents were not in business (M = 21.5, SD = 5.6) at t = 2.31, p <0.05. No other gender differences were found across other socio-demographic variables (p <0.05).

**Conclusion:**

Entrepreneurial Intentions differed among females with a history of business ownership. We argue that the complex interplay of gender demographic gaps in shaping undergraduate students’ entrepreneurial aspirations cannot be significantly improved based on family background without conclusive equal opportunity training approaches. Designing a hands-on and role-modeling entrepreneurship curriculum can shape gender-mainstream intentions.

## Background

Gender dynamics within entrepreneurship have gained increasing attention in recent years, yet the nuances of how gender influences entrepreneurial intentions continue to evolve with various dynamics. Whereas gender tends to be one of the socio-cultural variables determining perceptions and intentions of entrepreneurship ( Arora & Jain, 2019), males and females differ in their socio-cultural experiences and processes. It happens that the sexes may differ in their intentions to become entrepreneurs. Therefore, as students begin to develop entrepreneurial skills with high levels of training, the motivational goal is to lead a better-differentiated future life ( Korede & Badejo, 2021). Consequently, students maneuver through different school levels with the optimism of obtaining employment after school or college.

Notwithstanding, globally, the number of male entrepreneurs exceeds that of females, for 55–60% of entrepreneurial activity compared to women’s 40–45% ( Elam et al., 2019; Camelo-Ordaz, Diánez-González, & Ruiz-Navarro, 2016; Van Ewijk & Belghiti-Mahut, 2019). In some countries such United Arab Emirates (UAE), only 5.6% of reputable entrepreneurs and 9.0% of people who had enterprises as old as 3.5 years were females. Conversely, in Africa, the gender gap is narrower yet, disparities persist as women make up about 43% of entrepreneurs in Sub-Saharan Africa, the highest regional share worldwide, but their businesses are often smaller in scale and less growth-oriented (Global Entrepreneurship Monitor (GEM), 2022-2023). Female entrepreneurs own smaller enterprises that are less oriented towards growth than their male counterparts ( Van Ewijk & Belghiti-Mahut, 2019). These differences do not originate from women’s inability to have inner business potential or from inherent female weaknesses, such as deficiencies in networking experience or management skills rather, their inability, probably coupled with lower EIs, socio-cultural norms and perceptions as by individual skills and motivations.

Nonetheless, capability and ability differ based on socio informal and economic background, enterprise ownership, and in EIs ( Van Ewijk & Belghiti-Mahut, 2019). In specific aspects of particular communities, gender background in training institutions remains unexplored. For instance, the levels at which females and males are, with particular intentions to begin and manage new enterprises, is not well understood. Conversely, it was established that sex differences in EIs and entrepreneurial experience were common among both males and females ( Shneor & Jenssen, 2014). According to Kumar, Paray, and Dwivedi (2021), male students have higher perseverance towards individual entrepreneurial orientations and EIs. These discrepancies in the analysis of the phenomenon call for an in-depth analysis of the main influencing factors in the gendered entrepreneurship puzzle by focusing on the females and males undergoing entrepreneurship skills that this study is deemed to offer.

Notwithstanding, reports by the World Bank show that about 661,129 Ugandan youth enter the labor market annually, although this number is likely to double in the future, and no more than 20% of these will be employed in the formal sector ( Kyatusiimire, 2020). At the same time, Uganda faces youth unemployment, which increased from 1.93% in 2018 to 2.94% in 2021 ( World Bank, 2022). Additionally, universities are under increasing pressure to prepare graduates who can generate self-employment rather than wait for scarce wage jobs. Uncertainty surrounds the majority of youth’s odds of getting jobs in the formal sector if they are not ready to start up their own enterprises. Moreover, it is possible that a good number of those youth who get formal employment will be under-remunerated, hence making starting a personal enterprise a viable option for their financial prosperity. 

To address this trend, institutions need to send out graduates who can create self-employment. Indeed, entrepreneurship constitutes one of the major ways of solving unemployment-related challenges ( Izzuwan, Suhaila, & Sharafie, 2018; Grainca, 2022). Scholars have widely studied entrepreneurial initiatives, factors that affect entrepreneurship, and the influence of social norms on entrepreneurial decisions ( Pham, 2020; Efrata, Dwi Radianto, & Effendy, 2021; Bui, Nguyen, Tran, & Nguyen, 2020). However, little is known about gender and entrepreneurial intentions among undergraduate finalist students, especially in universities in African developing countries. Scholarly knowledge access remains unclear on whether male or female students who leave university are conscious of the challenge of unemployment or underemployment, and therefore intend to start their enterprises. Additionally, this study seeks to understand how entrepreneurial intentions differ among male and female finalists in high institutions of learning, specifically the Mbarara University of Science and Technology.

In this study, gender is considered an important dimension of entrepreneurship and education. Accordingly, entrepreneurial intention denotes the desire and commitment to initiate or improve an enterprise ( Korede & Badejo, 2021), or a person’s desire to start the enterprise ( Shahid, Imran, & Shehryar, 2018). This is especially true in the arena that underscores gender equity in financial independence. As a result, several studies have considered the impact of gender on entrepreneurial intentions and found stronger intentions among males ( Xanthopoulou & Sahinidis, 2022). Based on this background, the first objective proposal was to establish gender differences in entrepreneurial intentions among finalist undergraduate students.

Further, a wide spectrum of researchers considers that male students possess higher EIs, as entrepreneurship is widely regarded as a definite masculine adventure ( Arora & Jain, 2019; Kumar, Paray, & Dwivedi, 2021). While Arora and Jain (2019) found EI among male and female students, with males having higher EIs than females, limited information is available on the major factors that lead to gender-based influences on entrepreneurial intentions. This variation points to a critical research gap in understanding how gender operates in specific socio-economic and educational contexts, such as Ugandan universities.

To fill this gap, the paper draws on two complementary theoretical lenses. The theory of Planned Behaviour (TPB) ( Ajzen, 1991) emphasises the role of attitudes, subjective norms, and perceived behavioural control in shaping intentions, providing a structured way to examine how male and female students form entrepreneurial ambitions. The Family Systems theory ( Dore, 2008; Bandura et al., 2001) highlights how family functioning, socialisation, and support networks influence career development and entrepreneurial decision-making. Additionally, feminist liberal theory provides a critical perspective by questioning whether equal opportunities would translate into equal entrepreneurial intentions and by foregrounding the structural and cultural constraints that shape gendered choices ( Unger & Crawford, 1992; Jackson & Jones, 1998). Taken together, these perspectives allow this study to examine how individual psychology, family influence, and structural gender norms interact to shape entrepreneurial intentions among finalist undergraduate students at Mbarara University of Science and Technology. Additionally, by applying a multidimensional lens through interlinked theories, which together, provide a robust analytical framework that, debriefs how individual awareness, family structure, and gendered social contexts intersect to shape entrepreneurial intentions. Thus generating more insights in understanding the combined psychological, familial, and structural determinants of entrepreneurship among Ugandan university finalists.

## Gender variations and entrepreneurial intentions theoretical perspectives

Scholars in gender and entrepreneurship show that males possess stronger entrepreneurial intentions and skills than their female counterparts (e.g., Nikou, Brännback, Carsrud, & Brush, 2019). However, the trend is linked to deep-seated stereotypes within societies ( Gupta, Turban, Wasti, & Sikdar, 2009). This categorization places females in entrepreneurs’ masculine gender–role stereotypes. However, Karimi, Biemans, Lans, Chizari, and Mulder (2014) did not find gender differences in EIs; and there was no direct effect of gender on entrepreneurial intentions ( Smith, Sardeshmukh, & Combs, 2016). Gender entrepreneurial scholars have indicated that there are differences in various aspects of life, including skilling, environmental motivation, and higher levels of self-efficacy ( Nikou et al., 2019; Maes et al., 2014), although it is not clear which groups of individuals and groups of men and women were analyzed.

Therefore, this study will yield gender - comparative results for undergraduate students in the south, specifically the Mbarara University of Science and Technology. The study results are potentially helpful to universities in mitigating the likely wide entrepreneurial gap between boys and girls by focusing on increasing students’ beliefs about entrepreneurship intentions. Numerous studies have examined gender differences in entrepreneurial goals and identified subtle distinctions that influence the entrepreneurial environment of undergraduate finalist students. Studies by Dabic et al. (2012), Kossek et al. (2017), and Wilhau and Karau (2021) underscore the significant divergence in entrepreneurial aspirations between male and female students. Dabic’s findings suggest that male students often exhibit higher levels of self-efficacy and risk tolerance, contributing to a greater inclination; Kossek and Wu’s comprehensive analysis revealed that female students demonstrate equal levels of interest in entrepreneurship. However, their aspirations are less likely to translate into entrepreneurial activity because they frequently experience increased perceived constraints on family obligations, societal expectations, and resource accessibility ( Shastri, Shastri & Pareek, 2019). This delineation of intentions suggests a complex interplay of social, cultural, and psychological factors that influence the entrepreneurial trajectories of finalist students based on gender ( Kenye-Duma, 2022).

Moreover, the prior research has examined gendered variations in self-efficacy, risk tolerance, and motivation ( Dabic et al., 2012; Nikou et al., 2019; Maes et al., 2014), many of these studies are based on broad or unspecified populations, leaving unclear which groups of men and women were analysed. Similarly, while demographic factors such as age, socioeconomic status, and family obligations are recognised as influencing entrepreneurial aspirations ( Hechavarria & Ingram, 2016; Shastri, Shastri & Pareek, 2019), limited scholarship has systematically explored these dynamics in African higher education settings, mainly in Uganda. This creates a literature gap with limited empirical evidence on how gendered entrepreneurial intentions unfold among undergraduate finalist students in Uganda, particularly in regional universities such as Mbarara University of Science and Technology. Addressing this gap is essential for providing empirical insights into how social, cultural, and psychological factors interact to shape entrepreneurial aspirations, thereby enabling universities to take action to reduce the gender gap in entrepreneurship preparation.

Nonetheless, several demographic characteristics are closely linked to how gender affects entrepreneurial inclinations. Studies by Hechavarria and Ingram (2016) and Herrema (2017) highlight the intersectionality of gender with age, socioeconomic status, and educational background among finalist students. Hechavarria and Ingram’s study revealed that while both male and female finalist students initially showed enthusiasm for becoming entrepreneurs, over time, these intentions changed, with a high number of male students displaying persistence in pursuing business ventures. Additionally, Herrema’s analysis emphasizes the obstacles faced by female entrepreneurs ingrained between professionalism and cultural norms during the initial processes. This study further examines the evolution of entrepreneurial intentions among undergraduate boys and girls to concretize the contribution of training-based entrepreneurial intentions.

Although Family Systems theory identifies a widening entrepreneurial gap across demographic characteristics between boys and girls as influenced by family functioning, child development, and adaptation behaviors in Family Systems theory, other simulation factors, as identified by the Theory of Planned Behavior (TPB) ( Ajzen, 1991). The theory is based on the assumption that the proper performance of a human being is closely linked to self-efficacy and access to resources. For instance, male students may have easy access to resources and mobility, while female students may be limited because of competing domestic responsibilities. Therefore, TPB provides a structured lens to analyse how attitudes, social expectations, and perceived control interact with male and female students’ behaviors to explain the gendered variations in entrepreneurial intentions despite receiving similar education. This explains intentions through three components: 1) attitudes that reflect an individual’s beliefs about the outcomes of the behavior and their overall evaluation of those outcomes; 2) Subjective Norms, which involve the perceived social pressure or influence to perform or not perform, such as family, friends, or colleagues; and 3) Perceived Behavioral Control to an individual’s perception and ability to perform successfully.

These components provide a comprehensive framework for understanding and predicting entrepreneurial behavior across various demographic domains by undergraduate students in the Global South. According to Dore (2008), children’s behaviors and careers are influenced by the family system, which includes the way family members interact with one another, thus sustainably influencing them ( Bandura et al., 2001). Therefore, relevant to the phenomenon are individual attitudes towards entrepreneurship and how they are determined by perceived behavioural control over participation in various entrepreneurial activities.

In addition, the Succession Anxiety Theory ( Rowe et al., 2005) rests on assumptions that are guide the understanding intergenerational influences on entrepreneurial intentions by assuming that; leadership and ownership transitions evoke psychological tension and fear of loss among potential successor. Power, identity, and legacy are deeply intertwined, meaning that the thought of replacing a successful predecessor can trigger anxiety related to self-worth and role adequacy. The family and institutional settings often shape succession as both an emotional and structural process, where relational dynamics and expectations can either facilitate or constrain entrepreneurial initiative ( Shanine, Madison, Combs and Eddleston, 2023). Moreover, unmanaged succession anxiety can inhibit innovation and independent decision-making, as successors may avoid entrepreneurial ventures to escape perceived pressure ( Fries, 2024). The theory therefore integrate a theoretical basis for interpreting gendered differences in entrepreneurial motivation and guides in clarifying how intergenerational business contexts shape graduates entrepreneurship in higher education.

Furthermore, feminist liberal and structural perspectives ( Dobbs & Hamilton, 2007) are based on the assumption that equal training opportunities automatically translate into equal entrepreneurial intentions. Additionally, the theory views women as rational human beings who deserve the same human rights as men ( Tuana & Tong, 2018). Thus, gender justice is best achieved by modifying existing social institutions and other influential systems, such as political institutions. The theory is crucial in challenging the assumption that equal training opportunities ultimately translate into equal entrepreneurial intentions. While liberal feminist arguments highlight the importance of removing barriers so that women can exercise the same choices as men ( Unger & Crawford, 1992), broader feminist critiques stress that entrenched cultural norms and structural inequalities continue to shape women’s agency ( Jackson & Jones, 1998). Conversely, it emphasises that men and women have equal access to opportunities, rights, and resources, while recognising that persistent structural and cultural barriers often prevent women from achieving outcomes equal to men’s ( Johnson, 1991). These perspectives acknowledge both the promise of educational interventions and the persistent societal forces that may limit their impact. In this paper, different perspectives are applied to analyse and capture gender stereotypes, levels of access to capital, family expectations, and societal norms that shape women’s entrepreneurial choices, even when training is the same. Moreover, the theory guides in understanding that differences in entrepreneurial intentions are not simply a matter of individual motivation or capability, but are deeply embedded in broader gendered structures, thereby offering insights for designing more inclusive entrepreneurship programs and policies. This multifaceted approach not only clarifies why gender differences may emerge but also provides universities and policymakers with practical entry points to design interventions that foster more inclusive entrepreneurial ecosystems.

Therefore, the contention that if females have equal opportunities as men, their intentions would be similar ( Unger & Craw Ford, 1992) requires in-depth understanding through empirical analysis; however, feminists theorise and emphasize social differences that they persistently attribute to broader societal structures and cultural norms that shape gender roles and expectations ( Jackson & Jones, 1998). Nevertheless, all the theoretical perspectives are imperative for contributing to a deeper understanding of the nuanced dynamics influencing career choices in entrepreneurship and related impelling factors.

Furthermore, drawing from theoretical perspectives of planned behaviour coupled with feminism and family systems and Succession Anxiety Theory point of view, both individual psychological determinants and external environmental factors that influence entrepreneurial intentions in specific categories of female or male undergraduates are examined. This approach offers a more holistic understanding of entrepreneurial intentions by making it a valuable tool for analysing gender-based variations in entrepreneurial ambitions. We therefore, argue that multifaceted variations in entrepreneurial aspirations influence the persistence of gender inequality in the profession, and continue to limit the growth and promotion of entrepreneurial skills. This study provides valuable insights for educational institutions, curriculum developers, and policymakers.

This paper is structured to guide the reader from the broader context of gendered entrepreneurship to the specific realities of finalist undergraduate students at Mbarara University of Science and Technology. It begins with the Introduction and Background, which situates global and African gender disparities in entrepreneurship and highlights Uganda’s pressing youth unemployment challenge. The Theoretical Perspectives section which anchors the study in the theories by providing a multidimensional lens for analyzing entrepreneurial intentions. The Methods section outlining the design, sampling strategy, instrument adaptation, and analytical techniques applied to 404 students. The Results section presenting gender-disaggregated findings across socio-demographic characteristics and entrepreneurial intention scales, followed by the Discussion, which interprets the results in light of theory and literature, emphasizing the nuanced interactions of family background, cultural norms, and gendered experiences. The paper concludes with a Conclusion and Recommendations, offering implications for universities, policymakers, and curriculum developers to foster equitable entrepreneurial ecosystems.

## Methods

**The design:** The study design was cross-sectional and used a quantitative technique. The cross-sectional design enabled the collection of a large dataset within the limited time of the study. The quantitative approach worked in favour of analyzing a large amount of data and drawing inferences from the data within a short interval of time.

**The settings:** The study site was Mbarara University of Science and Technology (MUST). The university is located in Mbarara city, western Uganda. It has two campuses. The main campus is situated at Kihumuro Hill, seven kilometres on the Mbarara – Bushenyi – Fort Portal road, adjacent to Mbarara Stock Farm. The second and older campus of MUST is situated in the city centre, along Mbarara – Kabale highway. The second campus is approximately 269 kilometres southwest of the Ugandan capital city, Kampala.

**Sampling and sample size:** It consisted of finalist students taking business-related and educational programs. Finalist students were considered in the belief that they had already formed a self-concept in their respective programs of study. Moreover, they were at the summit of their respective programs and could easily think about what to do after campus. A total of 404 students were recruited from both faculties using a census. Including a pilot study, the study took place from May 2023 to December 2023. Ethical approval was received on 16 ^th^ October 2023 and participants were recruited into the study from October to December, 2023. Using a census was seen as a better strategy for taking a small population of students but also as a means of avoiding errors and bias associated with sampling.

**Procedure for data collection:** The study team sought approval from the Institutional Review Committee of the Mbarara University of Science and Technology. The team sought administrative clearance from the Deans of the participating faculties. The data collection exercise complied with COVID-19 standard operating procedures. Sampling took place in lecture rooms, mainly after the lectures. Consent was sought from the respondents, for example, assurance of confidentiality and freedom to withdraw from the study at any time, ensuring anonymity of participants. Each respondent was given a snack as a compliment to participate in this study.

**Instrument:** A self-administered questionnaire, consisting of sociodemographic characteristics and a modified Entrepreneurial Intentions (EIs) scale, was used for data collection. We modified the original EIs scale to adapt it to the Ugandan culture and context of students ( Lei & Lee, 2021). The initial scale item (e1) was: “have you ever seriously considered becoming an entrepreneur? Other items were added to the origial scale, to adjust for the realities in Ugandan context, for instance (e2): “have you ever seriously considered becoming an entrepreneur within one year after University?”; (e3): “have you ever seriously considered becoming an entrepreneur within five years after University?” (e4): “have you ever seriously considered becoming an entrepreneur more than five years after University?” The original scale options were retained: “1 = definitely not interested; 2 = Not interested; 3 = somewhat Un-interested; 4 = Cannot Say; 5 = Somewhat Interested; 6 = Interested; 7 = Extremely interested”. During exploratory factor analysis, all items on the new scale loaded onto Factor one, with loading over 0.4 - e1 loaded at 0.6712; e2 at 0.6184; e3 at 0.6672 and e4 at 0.5928 and the eigenvalue was over 1.0 (1.62963), implying the new scale was valid. The scale had an alpha reliability coefficient of 0.7132.

A self-administered questionnaire, consisting of sociodemographic characteristics and a modified Entrepreneurial Intentions (EIs) scale, was used for data collection. We modified the original EIs scale to adapt it to the Ugandan culture and context of students ( Lei & Lee, 2021). Students’ socio-demographic characteristics included gender, age, category of sponsorship, type of residence, history of business ownership, and number of parents. The selection of items on sociodemographic characteristics was based on literature review of common characteristics that influence EIs among college students ( Vuković, Jošanov-Vrgović, Jovin, & Papić-Blagojević, 2020). With those criteria in mind, several items regarding students’ characteristics were generated in the study instrument ( Vuković et al., 2020). We asked the students about their age in years. Participants were tasked to write their respective ages in a free space left for them in the questionnaire. Another item about whether they resided at campus (residents) or out of campus (non-residents), participants were tasked to tick “Yes” for residents and “No” for non-residents.

Further, an item was included about the type of sponsorship. It was on a three-point scale for “1 – Private sponsored”, “2 – Government sponsored” and “3 – Loan Scheme”. In Uganda’s higher education, privately sponsored students are those whose tuition fees are paid by their parents or by other private sponsors such as charity organizations. On the other hand, government-sponsored students are those with government scholarships awarded on merit during the joint university selection of the “best students”. Mainly, the “best students” in this case are those with top grades in terminal high school examinations. The third category, “loan scheme”, are students that qualify for university education but not among the top performers, and are not able to pay fees on a private basis. Such students apply for and obtain a “fees loan” from the government which they pay after graduating and joining the work industry. They are mainly students from the science disciplines.

The instrument also contained two items capturing participants’ fathers’/male guardian’s and mothers’/female guardian’s or family occupations/major source of income. Participants were requested to fill in the “occupations” in blank spaces. The responses were so diverse, ranging from informal occupations such as peasant farmers and retail traders to formal occupations such as health care practitioners and engineers. These responses regarding occupations were later dummied and coded into “business” and “other” occupations. The coding was to align the categories to the study aim and for clear interpretation.

More to the demographics were two items, each on a dichotomous scale. One of these items was regards to participants’ history of owning a business. The item sought to know whether the students ever owned a business of their own. Responses were either “1 - yes” or “2 - No”. Asking about history of business ownership was necessary as such history could potentially influence what students thought about entrepreneurship. The other item was as to whether a participant had parents/guardians or not. A participant that had no parents was required to select zero (“0”), while a participant with one or two parents was required to select “1”. This item was necessary, as parents’ activities and guidance could potentially influence entrepreneurial intentions.

As already mentioned, a modified EIs scale was used to measure entrepreneurial intentions ( Mirjana, Ana, & Marjana, 2018). The original scale had only one item: “Have you ever seriously considered becoming an entrepreneur?” on a Likert scale of 1 = definitely not interested, 2 = not interested, 3 = somewhat uninterested, 4 = cannot pay, 5 = somewhat interesting, 6 = interested, and 7 = extremely interested. Our modification of the scale included three extra items, but maintained the original likert e.g., “I intend to be an entrepreneur within 1 year after University.”

**Data management and analysis:** After data collection, data were entered into STATA 17 ( StataCorp, 2021) to check for missing values, outliers, and parametric assumption diagnostics to assess suitability for advanced analyses of the data. Descriptive and inferential statistics were used to analyze the data. Frequencies, percentages, and means were used in describing the samples. After descriptive analysis, t-tests of independent samples were used to compare EIs among gender and other demographic variables.

## Results

This study was designed to establish gender differences in entrepreneurial intention across the socio-demographic characteristics of finalist undergraduate students. Differences were analyzed using t-tests for independent samples. The results are recorded in the preceding tables.

Table 1 shows that of the 404 finalist undergraduate students enrolled in the majority were aged <25 years, 89.9% (n = 363) were aged < 25 years, with a mean age of 23.2 years (SD = 1.9). The majority were on private or government loans (80.4%, n = 325), non-residents (81.4%, n = 328), had no history of starting a business (80.4%, n = 325), and had at least one parent (97.8%, n = 395) ( Table 1). Most sociodemographic characteristics were statistically different across genders (p < 0.05), apart from the number of parents (p = 0.190), history of business ownership (p = 0.054), and male and female guardians’ occupations (p =0.670 and 0.160, respectively). Further results indicated that males (M = 23.4, SD = 1.9) were older than females (M = 22.9, SD = 2.1) (p < 0.01) ( Table 1).

**Table 1.** Socio-demographic characteristics of respondents (N = 404).

Characteristics	Category	Overall, n (%)	Males, 56.2% (n = 227)	Females, 43.8% (n = 177)	p
Age (years)	Mean± SD	23.2± 1.9	22.9±2.1)	23.4 ±1.7)	0.005 **
	<25	**363 (89.9)**	166 (93.8)	197 (86.8)	0.021 *
	≥25	41 (10.1)	11 (6.2)	30 (13.2)	
Sponsorship	Private/Loan	**325 (80.4)**	165 (93.2)	160 (70.5)	<0.001 ***
	Government	79 (19.6)	12 (6.8)	67 (29.5)	
Residence	Non-resident	**328 (81.2)**	136 (76.8)	192 (84.6)	0.048 *
	Resident	76 (18.8)	41 (23.2)	35 (15.4)	
History of business ownership	No	**325 (80.4)**	150 (84.7)	175 (77.1)	0.054
	Yes	79 (19.6)	27 (15.3)	52 (22.9)	
Number of parents	0	9 ( 2.2)	2 (1.1)	7 ( 3.1)	0.190
	≥1	**395 (97.8)**	175 (98.9)	220 (96.9)	
Male guardian’s occupation	Business	**192 (47.5)**	82 (46.3)	110 (48.5)	0.670
	Other	212 (52.5)	95 (53.7)	117 (51.5)	
Female guardian’s occupation	Business	**255 (63.1)**	105 (59.3)	150 (66.1)	0.160
	Other	149 (36.9)	72 (40.7)	77 (33.9)	

* p < 0.05.

** p < 0.01.

*** p < 0.001.

Table 2 shows that there were no statistically significant gender differences across the age groups. Although non-significant, the EIs showed a slight decrease across age for both female and male students. No significant sex differences were identified in the type of sponsorship. However, females on government sponsorship had slightly higher EIs than those on private sponsorship. As for student residences, female residents had slightly higher EIs than non-residents. However, the results were not statistically significant.

**Table 2.** Gender differences in entrepreneurial intentions among finalist undergraduate students (N = 404).

Characteristics	Category	M±SD	T	P	Females (n = 177)			Males (n = 227)	t	P
					M±SD	T	P	M±SD		
Age (years)	<25	20.4±6.4	0.84	0.404	20.1±6.7	0.42	0.677	20.7±6.2	0.85	0.394
	≥25	19.5±6.5			19.2±8.3			19.6±7.0		
Sponsorship	Private/Loan	20.2±6.8	0.48	0.635	19.9±6.9	0.70	0.482	20.5±6.6	0.06	0.948
	Government	20.6 ±5.5			20.3±4.3			20.5±5.7		
Residence	Non-resident	20.2±6.5	0.13	0.900	19.8±6.7	0.60	0.550	20.6±6.4	0.35	0.724
	Resident	20.4 ±6.6			20.6±7.0			20.2±6.3		
History of business ownership	No	20.1±6.6	0.98	0.330	19.5±6.9	2.25	**0.026** *	20.7±6.4	0.62	0.534
	Yes	20.9±6.0			22.7±5.1			20.0±6.3		
Parents	0	20.8 ±7.3	0.22	0.822	24.5±4.9	0.95	0.345	19.9±7.8	0.34	0.733
	≥1	20.3±6.5			20.0±6.8			20.5±6.3		
Male guardian’s occupation	Business	19.9 ±6.8	1.29	0.196	20.3±6.7	0.52	0.601	19.5±6.9	2.31	**0.022** *
	Other	20.7±6.2			19.8±6.8			21.5±5.6		
Female guardian’s occupation	Business	20.3±6.6	0.03	0.973	20.2±6.8	0.57	0.566	20.3±6.6	0.68	0.494
	Other	20.3±6.3			19.7±6.8			20.9±5.9		

* P < .05.

Statistically significant gender differences were found only across the history of business ownership among the female students (p <0.05). Females with a history of business ownership reported higher EIs (M = 22.7, SD = 5.1) than their counterparts with no history of business ownership (M = 19.5, SD = 6.9) at t =2.25, p < 0.05. Further, when male students had a male caretaker or parent with an occupation as business, they reported lower entrepreneurial intentions (M = 19.5, SD = 6.9) than their counterparts whose parents were not in business (M = 21.5, SD = 5.6) at t = 2.31, p < 0.05. No other differences in sex were found across other sociodemographic characteristics (p < 0.05).

## Discussion

This is a gender-based study using t-tests of independent samples to investigate Entrepreneurial Intentions across the demographic characteristics of undergraduate students at the University of Science and Technology. Statistically significant results were found among girls with regard to the history of owning a business and among boys with regard to male guardians’ occupation. Specifically, EIs were higher among females with a history of business ownership than females without a history of business ownership. The results are somewhat explainable in the theoretical arena. For instance Feminist Liberal Theory contends for equality of both girls and boys as defined by the training and exposure background ( Unger & Craw Ford, 1992). Girls show more interest in initiating their firms if they have been exposed to entrepreneurship through family or other sources. Similarly, boys who have grown up with men as guardians may be more inclined to view entrepreneurship as a feasible career route, although the results for boys are not significant. However, According to Bullough (2022), the obstacles that female entrepreneurs usually face are ingrained between professionalism and cultural norms during the initial processes, although the findings challenge them and shed light on the crucial role of experience in fostering entrepreneurial aspirations among girls.

Conversely, it is commonly perceived that greater barriers are related to family responsibilities, societal expectations, and resource accessibility, which eventually changes the entrepreneurial desires and focus of young boys and girls. Thus, it is likely that entrepreneurial action ( Jain & Ali, 2013). Therefore, the distinction between male and female goal points is a nuanced interaction between psychological and association-related elements that affects their entrepreneurial paths as finalists ( Kenye-Duma, 2022).

On the other hand, the Family Systems Theory affirms that when boys and girls share the same history of family background, entrepreneurial intentions should portray a sense of equality in choices ( Titelman, 2012). The results indicate gender gaps in relation to those with and without a history of family business ownership, which emphasizes the potential impact of practical engagement in shaping one’s inclination towards entrepreneurship. This emphasizes the need for educational institutions to create an environment that fosters experiential learning and provides platforms for students to engage in entrepreneurial endeavors ( Tomy & Pardede, 2020), ultimately contributing to shaping their career paths and aspirations. These insights emphasize the importance of offering students the opportunity to participate in entrepreneurial practices and gain related skills regardless of gender orientation.

Nonetheless, the findings indicate that male students with male guardians engaged in business reported lower entrepreneurial intentions. Through the lens of Succession Anxiety Theory ( Rowe et al., 2005; Lansberg, 1988) It can be meaningfully interpreted that the anticipation of inheriting or succeeding in a family enterprise evokes psychological tension, fear of inadequacy, and loss of autonomy among potential successors. Within the context of these results, such dynamics may explain why some male students, despite their exposure to entrepreneurial environments, expressed weaker entrepreneurial aspirations. The presence of a successful male guardian in business could heighten upward social comparison and anxiety about meeting established family expectations, thereby suppressing individual entrepreneurial drive and self-efficacy ( Shanine, Madison, Combs and Eddleston, 2023). Moreover, concerns over legitimacy and potential conflict in assuming leadership of a family business may discourage self-initiated entrepreneurial ventures, leading to withdrawal from entrepreneurial pathways. Note withstanding, findings suggest that exposure to family entrepreneurship does not uniformly enhance entrepreneurial intentions; rather, it can also generate identity and legacy pressures that constrain initiative particularly, among male students socialized to align with paternal success models ( Fries, 2024). Thus, emotional, relational, and psychological factors within family business settings shape the entrepreneurial outlook of young adults, underlining the need for mentorship and training programs that promote autonomy-supportive succession and reduce intergenerational anxiety in entrepreneurship education.

Furthermore, related to family history is the discernable relationship between the occupation of the guardians and the entrepreneur’s intentions. For instance, a male guardian was an influencing factor in determining whether someone would become an entrepreneur, and vice versa. Entrepreneurial intentions, therefore, were lower among male students whose male caretakers or parents had business as an occupation than their counterparts whose male parents were not in business. An indication that while the guardian’s entrepreneurship practices can enhance the perceived desirability and feasibility of entrepreneurship, it may also inhibit the translation of perceptions into actual career intentions due to upward social comparison mechanisms ( Criaco et al., 2017). Therefore, the Family System Theory holds ( Titelman, 2012) the presence of a parent/guardian role model in entrepreneurship can increase educational and training aspirations and expectancy for an entrepreneurial career ( Zubairu, 2022).

Conversely, Polin et al. (2016) indicated that the impact of parents’ careers on their children’s entrepreneurial intentions can also vary by gender, with self-employed mothers having a greater positive impact on their children’s intentions than self-employed entrepreneurial fathers. This implies that the gender of the parents involved in entrepreneurship may have highly differing but significant influence on their children’s aspirations in the field. These aspirations are thus attributed to the levels of parents’ socialization, where mothers tend to demonstrate a more sensitive and responsive character, whereas fathers tend to be more directive ( Ladd & Kochenderfer-Ladd, 2019). Therefore, the widening entrepreneurial gap across demographic characteristics between boys and girls is further described by the entrepreneurship-integrated model, as indicated in Entrialgo and Iglesias (2016) who analyzed the complements that emphasize highlighting the obstacles faced by female entrepreneurs as ingrained between professionalism and social and cultural norms mainly during the initial processes ( Herrema, 2017). The Entrepreneurship Integrated Model, which amalgamates various gendered differences in entrepreneurship intentions, such as psychological and feminist theories, ultimately advocates for more inclusive and equitable pathways in entrepreneurship. Therefore, an integrated model that considers the experience of family background recognizes the influence of social norms, encompassing societal expectations, cultural values, and role models, in shaping and reinforcing entrepreneurial intentions ( Entrialgo & Iglesias, 2016). In essence, the model catalyzes a holistic understanding of entrepreneurship intentions, paving the way for a more equitable and inclusive entrepreneurial landscape than being limited to training and historical experience.

## Conclusion

The study results deepen our understanding of how familial background, parental occupation, and gender dynamics influence entrepreneurial intentions. They offer insightful analyses of the complexities surrounding career decisions, stressing the significance of considering cultural norms, psychological factors, and familial influences when forming students’ goals for entrepreneurship. Specifically, EIs differed across females with a history of business ownership. Most importantly, having a parent or guardian as a role model in entrepreneurship generally encourages finalist students, both male and female, to aspire towards entrepreneurial careers. Although the gender of the parent involved in entrepreneurship showed a possible significant influence on entrepreneurial intentions, it had varying influence on their children’s intentions due to socialization effects.

The research findings are important in providing insights that guide designing of focused interventions, for instance by creating an atmosphere of more encouraging for aspiring entrepreneurs, regardless of their gender or family. Understanding these correlations can also inform the development of targeted educational interventions, mentorship programs, and support networks aimed at cultivating and empowering entrepreneurial ambitions among female students. For instance, promoting strategies for acquiring entrepreneurial experiences and providing the necessary resources and guidance to students could potentially foster a more inclusive entrepreneurial environment. Therefore, the nuanced interaction between association-related elements that influence entrepreneurial paths, such as family backgrounds, carries prospects for an institutional curriculum focus that encourages experiential learning with equal opportunities for all students. We argue that the complex interplay of gender demographic gaps in shaping undergraduate students’ entrepreneurial aspirations cannot be significantly improved based on family background without conclusive equal opportunity training approaches. Such approaches embrace mainstream gender equality skilling and equal socio-economic enhancement, purposely addressing the unique challenges and boosting the prospects of individuals with different backgrounds.

### Recommendations

To fill the demographic characteristics and gender gaps in the entrepreneurial initiatives of undergraduate students at the University of Science and Technology, the need to advocate for policies and initiatives at institutional and governmental levels that support and encourage entrepreneurship among students is important. This can be imperative by ensuring equal opportunities and resources for aspiring entrepreneurs of all sexes and backgrounds. Since most of the overarching demographic characteristics of finalist students indicate the experience to be the most influential in entrepreneurial initiatives, this has policy implications. This paper recommends a policy that considers various ways to promote entrepreneurship among all demographic groups, including girls and students from families without a history of entrepreneurship.

Furthermore, undergraduate students’ aspirations to become entrepreneurs in a gendered equal perceptive require a nurtured gender mainstreamed and amplified curriculum tailored to facilitate access to such experiences, particularly for female cohorts who may have historically encountered obstacles or cultural biases against entrepreneurship. For instance, designing a hands-on and role-modeling entrepreneurship curriculum caters for mainstream gender. Additionally, exploring how EIs’ intentions translate into actual entrepreneurial actions and success in the long term would provide valuable insights into the trajectory of aspiring entrepreneurs beyond their undergraduate years. These approaches enhance entrepreneurship intentions adopted from cultural and family backgrounds and simultaneously introduce skills to males and females that lack business background traces.

#### Ethical approval and consent

The study obtained ethical approval on 16/10/2023 from Mbarara University Ethical Committee (MUST -IREC), reference MUST – 2023 – 1067 from 16/10/2023-16/10/2024. Participants provided written informed consent using the official, approved MUST – IREC consent form.

## Data Availability

Figshare: Gender Variations and Entrepreneurial Intentions: A Cross-Sectional Analysis of Finalist Undergraduate Students at Mbarara University of Science and Technology. https://doi.org/10.6084/m9.figshare.26130826.v1 ( Rukundo et al., 2024a) This project contains the following files:
• Raw data Figshare: Questionnaire for the data regarding Gender Variations and Entrepreneurial Intentions: A Cross-Sectional Analysis of Finalist Undergraduate Students at Mbarara University of Science and Technology. https://doi.org/10.6084/m9.figshare.27134763 ( Rukundo et al., 2024c) Figshare: Informed consent regarding Gender Variations and Entrepreneurial Intentions: A Cross-Sectional Analysis of Finalist Undergraduate Students at Mbarara University of Science and Technology. https://doi.org/10.6084/m9.figshare.27135054 ( Rukundo et al., 2024b) This project contains the following files:
• Questionnaire • Consent form

## License

The underlying and extended data associated with this article are available under the terms of the Creative Commons Attribution 4.0 International License (CC-BY-4.0).
