# Supplementary material for: Gender Variations and Entrepreneurial Intentions: A Cross-Sectional Analysis of Finalist Undergraduate Students at Mbarara University of Science and Technology, Uganda
Source: F1000Res. 2025 Nov 13;14:136. Originally published 2025 Jan 27. [Version 2] doi: 10.12688/f1000research.148994.2 (PMC12749555; doi:10.12688/f1000research.148994.2)
Supplement: Peer review history

# Reviewer report: Reviewer response for version 2

**DOI:** 10.5256/f1000research.190101.r432354

**Published:** 2025-12-29

**Authors:** Juan Felipe Espinosa-Cristia

**Affiliations:** 1 Department of Ingeniería Comercial, Universidad Tecnica Federico Santa Maria, Valparaíso, Valparaiso Region, Chile

Based on the Version 2, that is your revised manuscript and the specific points that I raised in my previous peer review report, I have conducted a re-evaluation of the article "Gender Variations and Entrepreneurial Intentions: A Cross-Sectional Analysis of Finalist Undergraduate Students at Mbarara University of Science and Technology, Uganda." It is my opinion that the authors have made substantial efforts to address the reviewer's concerns, particularly regarding the theoretical depth of the discussion and the transparency of the methodological instruments.

 The most critical issue that I raised previously concerned with the reproducibility of the study due to the absence of the specific items used in the "modified Entrepreneurial Intentions (EIs) scale." IN this vein, the authors have successfully rectified this in their methods section. Furthermore, the authors explicitly listed the original item alongside the three added items regarding timeframes (one year, five years, and more than five years after university), the measurement of the dependent variable is now transparent. Such an addition permits the replication of the study and clarifies exactly how the authors operationalized "intention" beyond a simple binary desire, thereby satisfying the requirement for sufficient methodological detail.

 Regarding the statistical analysis, the authors have responded to the request to report on assumption checks. The revised text in the "Data management and analysis" section now is explicit about author's parametric assumption diagnostics that were conducted to assess suitability for advanced analyses. While the authors confirm that these checks were performed, a higher level of rigor would have been achieved by briefly reporting the specific outcomes of tests for normality (e.g., Shapiro-Wilk) or homogeneity of variances (e.g., Levene's test) rather than simply stating they were done. Nevertheless, the explicit acknowledgment of these diagnostic procedures addresses the reviewer's core concern regarding the validity of using t-tests, and given the sample size of 404, the robustness of the t-test against minor violations of normality is a valid implicit assumption.

 All in all, for me, the most significant improvement in this version it is at the core of the discussion section. The discussion about the counter-intuitive finding that male students with fathers in business exhibit lower entrepreneurial intentions is a nice addition to this article. The authors have integrated the Succession Anxiety Theory effectively to explain this phenomenon. The new text articulates how the anticipation of inheriting a family enterprise can evoke psychological tension, fear of inadequacy, and upward social comparison. By incorporating concepts such as "legitimacy" and "autonomy," the authors have elevated the paper from a descriptive study to one with meaningful explanatory power. This theoretical expansion aligns perfectly with the reviewer’s suggestion and turns a puzzling data point into a compelling contribution to the literature on family business succession and gendered entrepreneurship.

 The Background section has also been restructured to follow the "funnel" approach requested. The authors' text now logically flow from global gender dynamics in entrepreneurship to the specific economic context of Uganda, and finally anchors the study in the Theory of Planned Behavior and Family Systems Theory. This creates a more coherent narrative arc that justifies the research gap. However, the authors should ensure a final round of proofreading is conducted, as the new additions contain minor grammatical errors. For instance, in the Discussion section, the phrase "Note withstanding" appears where "Notwithstanding" was likely intended, and there are minor punctuation inconsistencies surrounding the citations of the new theories.

 In conclusion, I do think that the authors have comprehensively addressed the significant reservations outlined in the first peer review. The inclusion of the scale items ensures reproducibility, the theoretical discussion is now robust and insightful, and the structural flow of the introduction is improved. The manuscript now meets the scientific standards expected for indexing, provided the minor typographical errors introduced during the revision are corrected. I would recommend changing the status to "Approved"

Is the work clearly and accurately presented and does it cite the current literature?

Partly

If applicable, is the statistical analysis and its interpretation appropriate?

Partly

Are all the source data underlying the results available to ensure full reproducibility?

Yes

Is the study design appropriate and is the work technically sound?

Yes

Are the conclusions drawn adequately supported by the results?

Yes

Are sufficient details of methods and analysis provided to allow replication by others?

No

Reviewer Expertise:

Organization Studies, Social Sustainability, and Technology and Organizations.

I confirm that I have read this submission and believe that I have an appropriate level of expertise to confirm that it is of an acceptable scientific standard.

# Reviewer report: Reviewer response for version 2

**DOI:** 10.5256/f1000research.190101.r439788

**Published:** 2025-12-08

**Authors:** Juan P. Perez

**Affiliations:** 1 EAFIT School of Economics and Finance, Medellín, Antioquia, Colombia

Based on the Version 2, that is your revised manuscript and the specific points that I raised in my previous peer review report, I have conducted a re-evaluation of the article "Gender Variations and Entrepreneurial Intentions: A Cross-Sectional Analysis of Finalist Undergraduate Students at Mbarara University of Science and Technology, Uganda." It is my opinion that the authors have made substantial efforts to address the reviewer's concerns, particularly regarding the theoretical depth of the discussion and the transparency of the methodological instruments.

 The most critical issue that I raised previously concerned with the reproducibility of the study due to the absence of the specific items used in the "modified Entrepreneurial Intentions (EIs) scale." IN this vein, the authors have successfully rectified this in their methods section. Furthermore, the authors explicitly listed the original item alongside the three added items regarding timeframes (one year, five years, and more than five years after university), the measurement of the dependent variable is now transparent. Such an addition permits the replication of the study and clarifies exactly how the authors operationalized "intention" beyond a simple binary desire, thereby satisfying the requirement for sufficient methodological detail.

 Regarding the statistical analysis, the authors have responded to the request to report on assumption checks. The revised text in the "Data management and analysis" section now is explicit about author's parametric assumption diagnostics that were conducted to assess suitability for advanced analyses. While the authors confirm that these checks were performed, a higher level of rigor would have been achieved by briefly reporting the specific outcomes of tests for normality (e.g., Shapiro-Wilk) or homogeneity of variances (e.g., Levene's test) rather than simply stating they were done. Nevertheless, the explicit acknowledgment of these diagnostic procedures addresses the reviewer's core concern regarding the validity of using t-tests, and given the sample size of 404, the robustness of the t-test against minor violations of normality is a valid implicit assumption.

 All in all, for me, the most significant improvement in this version it is at the core of the discussion section. The discussion about the counter-intuitive finding that male students with fathers in business exhibit lower entrepreneurial intentions is a nice addition to this article. The authors have integrated the Succession Anxiety Theory effectively to explain this phenomenon. The new text articulates how the anticipation of inheriting a family enterprise can evoke psychological tension, fear of inadequacy, and upward social comparison. By incorporating concepts such as "legitimacy" and "autonomy," the authors have elevated the paper from a descriptive study to one with meaningful explanatory power. This theoretical expansion aligns perfectly with the reviewer’s suggestion and turns a puzzling data point into a compelling contribution to the literature on family business succession and gendered entrepreneurship.

 The Background section has also been restructured to follow the "funnel" approach requested. The authors' text now logically flow from global gender dynamics in entrepreneurship to the specific economic context of Uganda, and finally anchors the study in the Theory of Planned Behavior and Family Systems Theory. This creates a more coherent narrative arc that justifies the research gap. However, the authors should ensure a final round of proofreading is conducted, as the new additions contain minor grammatical errors. For instance, in the Discussion section, the phrase "Note withstanding" appears where "Notwithstanding" was likely intended, and there are minor punctuation inconsistencies surrounding the citations of the new theories.

 In conclusion, I do think that the authors have comprehensively addressed the significant reservations outlined in the first peer review. The inclusion of the scale items ensures reproducibility, the theoretical discussion is now robust and insightful, and the structural flow of the introduction is improved. The manuscript now meets the scientific standards expected for indexing, provided the minor typographical errors introduced during the revision are corrected. I would recommend changing the status to "Approved".

Is the work clearly and accurately presented and does it cite the current literature?

Yes

If applicable, is the statistical analysis and its interpretation appropriate?

Yes

Are all the source data underlying the results available to ensure full reproducibility?

Yes

Is the study design appropriate and is the work technically sound?

Yes

Are the conclusions drawn adequately supported by the results?

Yes

Are sufficient details of methods and analysis provided to allow replication by others?

Yes

Reviewer Expertise:

NA

I confirm that I have read this submission and believe that I have an appropriate level of expertise to confirm that it is of an acceptable scientific standard.

# Reviewer report: Reviewer response for version 1

**DOI:** 10.5256/f1000research.163389.r391816

**Published:** 2025-07-09

**Authors:** Juan Felipe Espinosa-Cristia

**Affiliations:** 1 Department of Ingeniería Comercial, Universidad Tecnica Federico Santa Maria, Valparaíso, Valparaiso Region, Chile

**Are sufficient details of methods and analysis provided to allow replication by others?**

 This is the most significant weakness of the manuscript. The authors claim that they used a "modified Entrepreneurial Intentions (EIs) scale," which is central to the entire study. However, they did not provide the full text of the cited modified scale. They mention that the original scale had one item and that their modification "included three extra items," but these items are not specified in the manuscript. Without the exact wording of the questions used to measure the primary dependent variable, replication is impossible.

**If applicable, is the statistical analysis and its interpretation appropriate?**

 The authors do not report whether the assumptions for the t-tests (e.g., normality of data, homogeneity of variances) were checked and met. While t-tests are robust to some violations, reporting on these assumptions is standard practice and enhances the rigor of the analysis. All in all, the interpretation of the statistically significant results is acceptable. However, the discussion of the most intriguing and counter-intuitive finding—that male students with fathers in business have lower entrepreneurial intention is underdeveloped. The authors briefly mention "upward social comparison mechanisms" but do not sufficiently unpack this concept. Why would seeing a successful parent in business lead to lower intentions? Is it fear of not measuring up? A desire to forge a different path? This finding is a major potential contribution of the paper and warrants a much deeper theoretical and contextual discussion. The current explanation is superficial and leaves the reader wanting more.

 This article demands some extra work to be indexed. When authors claim that there is a negative correlation between a father's business occupation and a son's EIs, they are touching on an interesting and novel point. The authors should expand this part of the discussion significantly. They could explore theories related to succession anxiety, identity formation, perceived pressure, or the specific nature of the parental businesses in the Ugandan context. This would state a more robust paper's contribution from a simple descriptive study to a more impactful explanatory one. 

 I also suggest revising the background section to create a more integrated "funnel," moving from the broad topic of gender and entrepreneurship to the specific challenges in Uganda, and then showing how the chosen theories (TPB, Family Systems) provide a lens to address a clearly articulated research gap.

Is the work clearly and accurately presented and does it cite the current literature?

Partly

If applicable, is the statistical analysis and its interpretation appropriate?

Partly

Are all the source data underlying the results available to ensure full reproducibility?

Yes

Is the study design appropriate and is the work technically sound?

Yes

Are the conclusions drawn adequately supported by the results?

Yes

Are sufficient details of methods and analysis provided to allow replication by others?

No

Reviewer Expertise:

Organization Studies, Social Sustainability, and Technology and Organizations.

I confirm that I have read this submission and believe that I have an appropriate level of expertise to confirm that it is of an acceptable scientific standard, however I have significant reservations, as outlined above.
